# Development and psychometric validation of a patient-centered Knowledge, Attitude, and Practice (KAP) Questionnaire for Geriatric Cancer Pain Management

**DOI:** 10.3389/fpsyg.2026.1808383

**Published:** 2026-09-09

**Authors:** Xiaoyan Li, Yubin Chen, Jihua Zou, Noor Mastura Mohd Mujar, Rohayu Hami, Nizuwan Azman

**Affiliations:** 1 School of Medicine, Lishui University, Lishui, Zhejiang, China; 2 Department of Community Health, Universiti Sains Malaysia, Pusat Kanser Tun Abdullah Ahmad Badawi (PKTAAB), Kepala Batas, Malaysia; 3 School of Nursing, Harbin Medical University, Daqing, Heilongjiang, China; 4 Department of Health Medicine, Nantong Institute of Technology, Nantong, Jiangsu, China; 5 Division of Research and Networking, Pusat Kanser Tun Abdullah Ahmad Badawi, Universiti Sains Malaysia, Kepala Batas, Malaysia

**Keywords:** cultural adaptation, geriatric cancer pain management, Knowledge-Attitude-Practice (KAP), psychometric validation, questionnaire development, supportive care

## Abstract

**Background:**

This study aimed to develop and psychometrically validate a patient-centered Knowledge–Attitude–Practice (KAP) questionnaire for cancer pain management in Chinese geriatric cancer patients. Existing pain assessment tools were largely developed for younger or mixed-age populations and seldom capture culture-specific psychosocial factors or the patient’s own perspective.

**Methods:**

The Knowledge–Attitude–Practice Questionnaire for Geriatric Cancer Pain Management (KAP-CPGQ) was developed across five sequential phases: item generation (literature review and semi-structured key-informant interviews with 6 clinical experts), content validity (a two-round modified Delphi consultation with an independent panel of 12 experts), face validity (cognitive interviewing with 10 geriatric cancer patients), construct validity, and reliability analysis. Construct validity was examined in 440 geriatric cancer patients, randomly partitioned into two independent subsamples (*n* = 220 each) for exploratory (EFA) and confirmatory factor analysis (CFA). Test–retest reliability was assessed in 30 participants after a 2-week interval.

**Results:**

The final instrument retained 28 items across three domains: knowledge (10 items), attitudes (9 items), and practices (9 items), each rated on a 5-point Likert scale. Content validity was supported by an overall content validity ratio (CVR) of 0.90 and a scale-level content validity index (S-CVI/Ave) of 0.91, and all 28 items exceeded the Item Impact Score threshold of 1.5 (range 3.5–4.2). EFA identified a three-factor structure (KMO = 0.94; Bartlett’s test *p* < 0.001) explaining 61.0% of the total variance, with all item loadings >0.50. CFA confirmed good model fit (χ^2^/df = 1.158; CFI = 0.986; NNFI = 0.984; IFI = 0.986; NFI = 0.903; AGFI = 0.869; RMR = 0.026; RMSEA = 0.027), with standardized factor loadings ranging from 0.66 to 0.83. Reliability was high: overall Cronbach’s α = 0.94 (subscales 0.92–0.93), split-half reliability = 0.90 (subscales 0.87–0.89), and 2-week test–retest reliability ranged from 0.89 to 0.91 across the three domains.

**Conclusion:**

The KAP-CPGQ is a reliable, valid, and culturally adapted instrument measuring knowledge, attitudes, and practices in geriatric cancer pain management. It offers clinicians and researchers a tool to identify patient-level barriers to pain control and to guide targeted educational and behavioral interventions for geriatric cancer patients.

## 1 Introduction

Population aging is fundamentally reshaping the global cancer landscape. According to GLOBOCAN 2022, adults aged 60 years and older account for approximately 65% of newly diagnosed cancer cases and over 72% of cancer-related deaths worldwide (Zheng et al., 2025). China is undergoing one of the fastest demographic transitions globally, with the proportion of adults aged ≥60 years rising from 13.3% in 2010 to 18.7% in 2020 and projected to reach 28% by 2040, accompanied by a concomitant increase in cancer incidence among older adults (Sung et al., 2021; Diao et al., 2025). Within this population, cancer-related pain is disproportionately concentrated. A 2023 systematic review and meta-analysis of 444 studies reported an overall prevalence of cancer pain of 44.5%, increasing to 55% during active anticancer therapy and reaching 66.4% among patients with advanced or metastatic disease (Snijders et al., 2023). In prospective geriatric oncology cohorts, nearly half of patients aged 70 years and older experience moderate-to-severe pain at diagnosis (Brunello et al., 2019).

Cancer pain in geriatric patients is clinically complex and multidimensional. It arises from nociceptive, neuropathic, and treatment-related mechanisms superimposed on a background of multimorbidity, frailty, sensory impairment, and polypharmacy (Hachem et al., 2019; Brant, 2018). This multidimensionality is associated with accelerated functional decline, depression, increased hospitalization, and marked deterioration in health-related quality of life (Tagliafico et al., 2025; Brant, 2018). Despite widespread implementation of the World Health Organization’s three-step analgesic ladder, decades of evidence indicate that older patients remain systematically undertreated compared to younger cohorts (Greco et al., 2014; Bernabei et al., 1998). Such undertreatment reflects the interaction of patient-, clinician-, and system-level barriers, including culturally ingrained stoicism, ageism, opioid-related fears, unequal access to palliative care, and inconsistent clinical practices (Darawad et al., 2019; Xu et al., 2018; Oldenmenger et al., 2009; Mercadante and Arcuri, 2007; Liu et al., 2023). Therefore, effective pain management in geriatric oncology is not merely a component of comfort care but a key determinant of survival, autonomy, and dignity.

Accurate assessment of pain in geriatric patients remains a significant challenge, as most widely used instruments were developed for younger or mixed-age populations. Pain assessment depends on patients’ ability and willingness to translate complex bodily experiences into structured responses (Herr, 2011), a process often hindered in geriatric patients by cognitive impairment, sensory decline, culturally embedded stoicism, beliefs that pain is an inevitable consequence of aging or cancer, clinician ageism, and atypical or attenuated pain presentations due to altered nociceptive processing (Xu et al., 2018; Herr, 2011; Schofield, 2018; Zeng et al., 2020; Lukas et al., 2013). Existing tools–including the Brief Pain Inventory (BPI; Cleeland and Ryan, 1994), the McGill Pain Questionnaire (MPQ; Melzack, 1975), and the Pain Attitudes Questionnaire-Revised (PAQ-R; Mah et al., 2017) –reflect cultural assumptions about pain expression, opioid use, and patient–clinician communication that are not directly transferable to Chinese geriatric oncology settings (Xu et al., 2023; Ngamkham et al., 2012).

Substantial progress has been made in the cross-cultural adaptation of pain assessment tools in China. Early work on the Chinese version of the BPI (BPI-C) demonstrated the feasibility of psychometric adaptation for cancer pain assessment (Wang et al., 1996). Subsequent decades have produced a growing portfolio of instruments validated primarily in non-cancer chronic pain populations, including the Simplified Chinese Graded Chronic Pain Scale-Revised (C-GCPS-R; Liang et al., 2023), the Simplified Chinese Pain Catastrophizing Scale (SC-PCS; Yap et al., 2008), the Chinese short-form McGill Pain Questionnaire-2 (Wang et al., 2017), the Chinese Pain Care Quality Surveys (C-Pain CQ; Guo et al., 2020), and the Chinese Breakthrough Pain Assessment Tool (BAT-C; Liu et al., 2025). These efforts have progressed from simple back-translation to rigorous psychometric programs guided by Brislin’s framework and COSMIN standards. Nevertheless, major gaps remain. Most validation studies have focused on non-cancer chronic pain, and few instruments adequately capture older adults’ knowledge, attitudes, and self-management behaviors regarding cancer pain.

The Knowledge–Attitude–Practice (KAP) framework offers a theoretically grounded and empirically validated approach to address this gap. First proposed in health education theory in the 1980s (Bettinghaus, 1986), the KAP framework posits that knowledge influences attitudes, which in turn shape behaviors (Kang and Bagaoisan, 2024; Andrade et al., 2020). Over the past two decades, it has been operationalized through validated instruments across various chronic and oncologic conditions, including tuberculosis among Chinese student patients (Andrade et al., 2020), chronic kidney disease (Tariq et al., 2022), post-percutaneous coronary intervention self-management (Lei et al., 2024), diabetes mellitus and hyperuricemia self-management (Wang et al., 2024), cardiovascular and cerebrovascular rehabilitation (Zhou et al., 2024), and gastric cancer prevention (Kang and Bagaoisan, 2024). Contemporary applications increasingly recognize patients–not solely clinicians–as the primary agents of chronic disease and pain self-management. However, in cancer pain research, KAP has been applied almost exclusively to healthcare providers’ knowledge and attitudes (Darawad et al., 2019; Bouya et al., 2019; Kasasbeh et al., 2017; Makhlouf et al., 2023), whereas patients’ KAP–especially among geriatric Chinese cancer patients influenced by traditional beliefs, opioid-related fears, and age-related stigma–remains largely unassessed using culturally adapted, validated instruments (Zeng et al., 2020; Chi et al., 2023; Cleeland, 1998).

Considering the high burden of cancer pain among older Chinese adults, the limited cultural and geriatric specificity of existing tools, and the lack of patient-centered KAP instruments, the present study aimed to develop and validate the Knowledge–Attitude–Practice Questionnaire for Geriatric Cancer Pain Management (KAP-CPGQ). Grounded in the KAP framework and informed by literature synthesis and culturally embedded clinical phenomena such as stoicism, opioid-related fear, and intergenerational caregiving, this instrument is designed to comprehensively evaluate geriatric Chinese cancer patients’ knowledge, attitudes, and practices in pain management. By providing a psychometrically robust and culturally relevant tool, this study seeks to enable systematic evaluation of patient-level determinants of pain control and to inform the development of targeted educational and behavioral interventions to improve quality of life in geriatric cancer patients.

## 2 Materials and methods

This study adheres to the Strengthening the Reporting of Observational Studies in Epidemiology (STROBE) guidelines and conforms to the ethical principles outlined in the Helsinki Declaration (2013).

### 2.1 Study design

This methodological study was conducted to develop and validate the Knowledge–Attitude–Practice Questionnaire for Geriatric Cancer Pain Management (KAP-CPGQ). The development process followed best practices for instrument design (Boateng et al., 2018) and was completed in five sequential phases: (I) item generation, (II) content validity assessment, (III) face validity evaluation, (IV) construct validity testing, and (V) reliability analysis.

#### 2.1.1 Phase I: item generation

A preliminary item pool was developed through a sequential, two-pronged methodology comprising (a) a comprehensive literature review and (b) semi-structured key-informant interviews with clinical experts.

(a) Comprehensive literature review

From June to September 2024, two researchers (XL, YC) independently searched four Chinese databases (CNKI, Wanfang, VIP, SinoMed) and four English databases (PubMed, EBSCO, Springer Link, ScienceDirect) using Boolean search strings covering cancer pain, pain management, knowledge, attitudes, practices, elderly or geriatric populations, and questionnaire, instrument, or scale. The search was limited to peer-reviewed publications dated from January 2000 to August 2024. The initial search identified 2,847 records (Chinese: 1,234; English: 1,613). After removing 891 duplicates, 1,956 records were screened based on titles and abstracts. Of these, 142 full-text articles were assessed for eligibility, and 24 studies met the inclusion criteria. These studies were subsequently analyzed to extract candidate constructs and preliminary items. Any discrepancies were resolved through consensus with a third researcher (NMM).

(b) Semi-structured expert interviews

Six clinical experts were purposively recruited from three tertiary hospitals. Eligible participants held senior professional titles (associate chief physician/nurse or higher), had ≥10 years of relevant clinical experience in oncology, geriatric medicine, pain medicine, or oncology nursing, and possessed prior experience in geriatric cancer pain management. The expert panel included two oncologists, one pain physician, one geriatrician, and two senior oncology nurses. Three sequential interview rounds were conducted in October and November 2024, with each expert participating in one session per round (six sessions per round; eighteen sessions in total). Sessions lasted 60–90 min (mean = 72 min) and were conducted in a hybrid format, including four in-person sessions and two via secure video conferencing (Tencent Meeting).

Each session followed a semi-structured interview guide with five core open-ended questions and follow-up probes. The guide covered five thematic domains aligned with the KAP framework and geriatric oncology pain: (1) knowledge gaps and misconceptions regarding cancer pain mechanisms, pharmacological treatment principles (e.g., WHO analgesic ladder, opioid titration, adverse-effect management), and age-appropriate pain assessment; (2) culturally embedded pain beliefs; (3) attitudinal barriers, including opioid-phobia and ageism; (4) self-management behaviors, including medication adherence and non-pharmacological strategies; and (5) limitations of existing assessment tools for older adults.

The study employed three rounds: Round 1 identified constructs, Round 2 refined items, and Round 3 consolidated the item pool. All sessions were audio-recorded, transcribed verbatim, and thematically analyzed (Braun and Clarke, 2006). Coding was performed independently by two researchers. Themes and codes from the interviews were triangulated with candidate constructs and draft items from the literature. Items were mapped onto the three KAP domains, duplicates removed, and reworded into plain language for older adults with limited health literacy. This process produced a preliminary item pool of 42 items: knowledge (14 items, B1–B14), attitudes (15 items, B15–B29), and practices (13 items, B30–B42).

Representative items developed during this phase demonstrate the integration of literature-derived constructs with culturally salient clinical phenomena identified by the expert panel through their cumulative practice experience with Chinese geriatric cancer patients. For example, the practice-domain item B31, “I adhere to a prescribed schedule for taking analgesic medications, rather than delaying administration until the pain intensifies” was developed in response to experts’ repeated observations that scheduled, around-the-clock analgesic dosing is frequently misunderstood by Chinese geriatric patients as unnecessary unless pain becomes intolerable. Similarly, the attitudinal item B16,“ I do not consider “enduring pain” to be a virtue for the elderly; expressing pain is an essential avenue for obtaining appropriate treatment” was designed to capture the culturally specific construct of stoicism, or ren tong (忍痛). Across all three interview rounds, experts consistently identified this belief as a major barrier to pain disclosure and timely analgesic use among older Chinese patients.

#### 2.1.2 Phase II: content validity

Content validity was established using a modified Delphi technique, following established methodological standards (Hasson et al., 2000; Boulkedid et al., 2011) and aligning with the COSMIN framework for content validity assessment (Terwee et al., 2018; Mokkink et al., 2010).

##### 2.1.2.1 Expert panel

A multidisciplinary panel of 12 specialists was recruited through purposive sampling. The panel was independent of the Phase I interview panel and comprised three medical oncologists, three pain medicine physicians, two geriatricians, three senior oncology nurses, and one psychometrician. All panel members held senior professional titles and had at least 10 years of relevant clinical or research experience.

##### 2.1.2.2 Rating instrument

Each item was independently and anonymously rated by every expert on four attributes: relevance, clarity, simplicity, and freedom from ambiguity. Ratings were recorded on a four-point Likert scale anchored uniformly across the four attributes as follows: 1 = the item does not meet the attribute; 2 = the item partially meets the attribute and requires major revision; 3 = the item largely meets the attribute and requires only minor revision; and 4 = the item fully meets the attribute and requires no revision. Mandatory free-text qualitative comments accompanied any rating below 4 to inform iterative item refinement

##### 2.1.2.3 Delphi round procedure

Two formal rounds of the Delphi process were conducted between November and December 2024. Each round constituted a complete cycle comprising: (i) independent and anonymous rating of all candidate items by each expert through email using an electronic questionnaire; (ii) statistical aggregation of the ratings; (iii) preparation of a controlled feedback report; and (iv) circulation of the feedback report together with revised items to inform the subsequent round. Round 1 evaluated the full 42-item pool. Round 2 re-evaluated only those items that had been revised, flagged for merging, or had failed to reach consensus in Round 1; items achieving stable consensus in Round 1 were carried forward unchanged.

##### 2.1.2.4 Statistical metrics

The Content Validity Ratio (CVR) was calculated using Lawshe’s (1975) formula, CVR = (n_e_ - N/2)/(N/2), where n_e_ is the number of experts rating an item as “essential” and N is the total number of expert raters. Item retention was based on the critical CVR values recalculated for the panel size (Wilson et al., 2012). For a panel of 12 experts, the critical CVR value was set at 0.62, following Ayre and Scally (2014). The Item-level Content Validity Index (I-CVI) was calculated separately for each attribute–CVI (Relevance), CVI (Clarity), CVI (Simplicity), and CVI (Freedom from Ambiguity)–as the proportion of experts assigning a rating of 3 or 4 (Lynn, 1986). The total CVI for each item (CVI Total) was calculated as the mean of the four attribute-level I-CVIs. The Scale-level Content Validity Index using the average method (S-CVI/Ave) was then calculated as the mean of all CVI Total values across items (Polit and Beck, 2006).

##### 2.1.2.5 *A priori* decision rules

To ensure objectivity and reproducibility, the following decision rules were pre-specified. Items were retained unchanged if they had a CVR ≥ 0.62 and an I-CVI ≥ 0.79 across all attributes, with no expert recommending revision; Items were retained with revision if these thresholds were met but two or more experts recommended wording or cultural adaptations; Item pairs were merged if they showed substantial conceptual overlap, were flagged by three or more experts, and had CVI values within ± 0.05 of each other; Items were deleted if they had a CVR < 0.62 or an I-CVI < 0.79 on any attribute, were redundant with a higher-scoring item, or addressed content outside the scope of geriatric cancer pain management.

#### 2.1.3 Phase III: face validity

##### 2.1.3.1 Participants

Face validity was evaluated in January 2025 through cognitive interviewing (Willis, 2004), with item-level retention guided by the Item Impact Score procedure (Juniper et al., 1997). Ten geriatric cancer patients were purposively recruited from the outpatient clinic of a tertiary A-level hospital. Inclusion criteria were: (i) pathologically confirmed malignancy; (ii) age ≥60 years; (iii) self-reported pain intensity ≥3/10 on the Numeric Rating Scale (NRS) within the preceding week; (iv) intact cognition, as indicated by a Mini-Mental State Examination score ≥24; and (v) voluntary participation with written informed consent. The sample (5 men and 5 women, aged 62–78 years) represented diversity in cancer type, educational background, and pain duration.

##### 2.1.3.2 Administration

Participants completed the 28-item draft questionnaire independently in a private consultation room, which required 18–25 min (mean = 21 min). Following completion, each participant engaged in a 15–20-min structured cognitive debriefing, during which they evaluated each item on three dimensions–clarity, comprehensibility, and importance–using 5-point Likert scales. The Item Impact Score was calculated as the product of the frequency of respondents rating an item ≥4 on importance and the mean importance score (Broder et al., 2007). Items achieving an Impact Score ≥1.5 were considered to demonstrate acceptable face validity.

#### 2.1.4 Phase IV: construct validity

A cross-sectional survey was conducted among geriatric cancer patients consecutively recruited from the oncology and pain management departments of three tertiary A-level hospitals in Zhejiang Province between January and May 2025. Inclusion criteria were: (i) pathologically or histologically confirmed malignancy; (ii) age ≥60 years; (iii) self-reported cancer-related pain within the preceding month; (iv) intact cognition (Mini–Mental State Examination score ≥ 24); and (v) voluntary participation with written informed consent. Patients were excluded if they had severe cognitive, sensory, or communication impairments precluding reliable self-report; were clinically unstable or had an estimated survival of less than 3 months; had concurrent non-cancer chronic pain of clinically significant severity; or had participated in earlier developmental phases of this study. Using Kendall’s rule of thumb (10–20 participants per item), a minimum of 420 responses was required; of 504 questionnaires distributed, 440 valid responses were obtained (effective rate: 87.3%) and randomly split into two independent samples (*n* = 220 each) for exploratory factor analysis (EFA) and confirmatory factor analysis (CFA), respectively.

Exploratory factor analysis was conducted using principal component analysis with varimax rotation to identify factor structures. Sampling adequacy was confirmed (KMO = 0.94, Bartlett’s test *p* < 0.001). Factors were retained based on eigenvalues >1.0 and theoretical interpretability. CFA was performed using AMOS 26.0 to confirm model fit, evaluated by χ^2^/df, AGFI, NFI, NNFI, CFI, IFI, RMR, and RMSEA indices.

#### 2.1.5 Phase V: reliability analysis

Internal consistency was evaluated using Cronbach’s α (≥0.70 acceptable) and split-half reliability for the full Phase IV sample (*n* = 440). Test–retest reliability was assessed in a randomly selected subsample of 30 participants drawn from the Phase IV cohort, who met the same inclusion and exclusion criteria, had clinically stable pain status with no planned change in analgesic regimen, and completed a second administration 2 weeks later–an interval chosen to minimize recall bias while precluding substantive change in the underlying KAP constructs. Data were analyzed using SPSS 26.0 and AMOS 26.0, with *p* < 0.05 considered statistically significant.

## 3 Results

### 3.1 Content validity

Across two rounds of the Delphi consultation, the initial pool of 42 items was reduced to a final set of 28. In Round 1, eight items were deleted, four items (two pairs) were identified for merging into two items, and two items (B1 and B5) were rephrased. In Round 2, two additional items were deleted, and four items were flagged for merging into two items. Of the 42 initially generated items, 28 were ultimately retained, comprising 22 verbatim items, 2 revised items, and 4 newly created items consolidated from 8 source items. Overall, 10 items were deleted (B11, B12, B23, B26–B29, and B39–B41), and 8 source items were merged into 4 final items (B3+B4, B6+B7, B19+B20, and B35+B36). The complete audit trail is presented in Table 1, and the full item-level disposition is provided in Supplementary Additional File 1. The final 28-item version achieved an overall content validity ratio (CVR) of 0.90 and an average scale-level content validity index (S-CVI/Ave) of 0.91, as reported in Table 2.

**TABLE 1:** Disposition of all 42 originally generated items after two-round Delphi consultation (*n* = 12 experts).

Original ID	Domain	Final ID	CVR	CVI (relevance)	CVI (clarity)	CVI (simplicity)	CVI (ambiguity)	CVI (total)	Disposition	Reason for action
B1	K	K1	0.83	0.92	0.90	0.88	0.93	0.91	Revised	Wording simplification for elderly readers
B2	K	K2	0.92	0.95	0.92	0.90	0.95	0.93	Retained	Met all thresholds
B3+B4	K	K3	0.83	0.88	0.90	0.85	0.92	0.89	Merged	Conceptual overlap on self-report assessment
B5	K	K4	0.83	0.90	0.88	0.85	0.90	0.88	Revised	Disambiguation of geriatric-specific complications
B6+B7	K	K5	0.83	0.92	0.88	0.90	0.93	0.91	Merged	Conceptual overlap on opioid safety
B8	K	K6	0.92	0.92	0.95	0.90	0.93	0.93	Retained	Met all thresholds
B9	K	K7	0.92	0.93	0.90	0.92	0.95	0.93	Retained	Met all thresholds
B10	K	K8	0.92	0.90	0.88	0.85	0.92	0.89	Retained	Met all thresholds
B11	K	–	**0.42**	**0.55**	**0.62**	**0.50**	**0.62**	**0.57**	Deleted	Excessive technical complexity for lay self-report
B12	K	–	**0.50**	**0.65**	**0.70**	**0.68**	**0.75**	**0.69**	Deleted	Conceptual redundancy with K6
B13	K	K9	0.92	0.95	0.92	0.88	0.93	0.92	Retained	Met all thresholds
B14	K	K10	0.92	0.88	0.92	0.90	0.95	0.91	Retained	Met all thresholds
B15	A	A11	0.92	0.95	0.90	0.92	0.88	0.91	Retained	Met all thresholds
B16	A	A12	0.92	0.90	0.93	0.88	0.92	0.91	Retained	Met all thresholds
B17	A	A13	0.92	0.92	0.88	0.90	0.95	0.91	Retained	Met all thresholds
B18	A	A14	0.92	0.88	0.90	0.92	0.90	0.90	Retained	Met all thresholds
B19+B20	A	A15	0.83	0.90	0.85	0.88	0.87	0.88	Merged	Conceptual overlap on patient-rights orientation
B21	A	A16	0.92	0.90	0.92	0.88	0.93	0.91	Retained	Met all thresholds
B22	A	A17	0.92	0.93	0.88	0.90	0.92	0.91	Retained	Met all thresholds
B23	A	–	**0.50**	**0.62**	**0.70**	**0.68**	**0.72**	**0.68**	Deleted	Limited cultural applicability; outside KAP framework
B24	A	A18	0.92	0.92	0.90	0.88	0.95	0.91	Retained	Met all thresholds
B25	A	A19	0.92	0.88	0.92	0.90	0.88	0.90	Retained	Met all thresholds
B26	A	–	**0.42**	**0.58**	**0.65**	**0.62**	**0.68**	**0.63**	Deleted	Conceptual redundancy with A11
B27	A	–	**0.50**	**0.62**	**0.68**	**0.65**	**0.70**	**0.66**	Deleted	Insufficient specificity to pain self-management
B28	A	–	**0.50**	**0.65**	**0.70**	**0.62**	**0.72**	**0.67**	Deleted	Conceptual redundancy with B30
B29	A	–	**0.42**	**0.55**	**0.60**	**0.58**	**0.65**	**0.60**	Deleted	Limited cultural applicability
B30	P	P20	0.92	0.92	0.90	0.88	0.93	0.91	Retained	Met all thresholds
B31	P	P21	0.92	0.90	0.92	0.88	0.90	0.90	Retained	Met all thresholds
B32	P	P22	0.92	0.88	0.90	0.92	0.88	0.90	Retained	Met all thresholds
B33	P	P23	0.92	0.93	0.88	0.90	0.92	0.91	Retained	Met all thresholds
B34	P	P24	0.92	0.90	0.88	0.92	0.90	0.90	Retained	Met all thresholds
B35+B36	P	P25	0.83	0.88	0.87	0.85	0.90	0.88	Merged	Conceptual overlap on activity self-regulation
B37	P	P26	0.92	0.88	0.92	0.90	0.88	0.90	Retained	Met all thresholds
B38	P	P27	0.92	0.90	0.88	0.92	0.90	0.90	Retained	Met all thresholds
B39	P	–	**0.50**	**0.65**	**0.72**	**0.68**	**0.74**	**0.70**	Deleted	Conceptual redundancy with P26
B40	P	–	**0.42**	**0.55**	**0.62**	**0.60**	**0.68**	**0.61**	Deleted	Insufficient specificity
B41	P	–	**0.50**	**0.62**	**0.70**	**0.65**	**0.72**	**0.67**	Deleted	Limited cultural applicability
B42	P	P28	0.92	0.92	0.88	0.90	0.93	0.91	Retained	Met all thresholds

K = Knowledge; A = Attitude; P = Practice. Bold red values indicate sub-threshold scores (CVR < 0.62 or any CVI < 0.79). Thresholds: CVR ≥ 0.62 (Lawshe critical value for *N* = 12; Ayre and Scally, 2014); I-CVI ≥ 0.79 (Lynn, 1986). CVI (Total) is the mean of the four attribute-level I-CVI values.

**TABLE 2:** Content validity and face validity of final 28-item KAP-Cancer Pain Geriatric Questionnaire.

Final ID	Sources item(s)	CVR	CVI (relevance)	CVI (clarity)	CVI (simplicity)	CVI (ambiguity)	CVI (total)	Impact score	Clarity (mean)	Comprehensibility (mean)
K1	B1 (Revised)	0.83	0.92	0.90	0.88	0.93	0.91	3.8	4.4	4.3
K2	B2	0.92	0.95	0.92	0.90	0.95	0.93	4.2	4.6	4.6
K3	B3+B4 (Merged)	0.83	0.88	0.90	0.85	0.92	0.89	3.6	4.2	4.2
K4	B5 (Revised)	0.83	0.90	0.88	0.85	0.90	0.88	3.7	4.3	4.2
K5	B6+B7 (Merged)	0.83	0.92	0.88	0.90	0.93	0.91	3.9	4.5	4.4
K6	B8	0.92	0.92	0.95	0.90	0.93	0.93	4.1	4.7	4.8
K7	B9	0.92	0.93	0.90	0.92	0.95	0.93	4.0	4.6	4.5
K8	B10	0.92	0.90	0.88	0.85	0.92	0.89	3.8	4.4	4.3
K9	B13	0.92	0.95	0.92	0.88	0.93	0.92	4.0	4.6	4.6
K10	B14	0.92	0.88	0.92	0.90	0.95	0.91	3.9	4.4	4.4
A11	B15	0.92	0.95	0.90	0.92	0.88	0.91	4.1	4.5	4.4
A12	B16	0.92	0.90	0.93	0.88	0.92	0.91	3.9	4.3	4.3
A13	B17	0.92	0.92	0.88	0.90	0.95	0.91	4.0	4.4	4.3
A14	B18	0.92	0.88	0.90	0.92	0.90	0.90	3.8	4.3	4.2
A15	B19+B20 (Merged)	0.83	0.90	0.85	0.88	0.87	0.88	3.6	4.0	4.0
A16	B21	0.92	0.90	0.92	0.88	0.93	0.91	3.9	4.4	4.4
A17	B22	0.92	0.93	0.88	0.90	0.92	0.91	4.0	4.5	4.4
A18	B24	0.92	0.92	0.90	0.88	0.95	0.91	3.9	4.6	4.7
A19	B25	0.92	0.88	0.92	0.90	0.88	0.90	3.8	4.3	4.3
P20	B30	0.92	0.92	0.90	0.88	0.93	0.91	3.9	4.4	4.4
P21	B31	0.92	0.90	0.92	0.88	0.90	0.90	3.8	4.3	4.2
P22	B32	0.92	0.88	0.90	0.92	0.88	0.90	3.7	4.3	4.2
P23	B33	0.92	0.93	0.88	0.90	0.92	0.91	4.0	4.5	4.5
P24	B34	0.92	0.90	0.88	0.92	0.90	0.90	3.8	4.4	4.3
P25	B35+B36 (Merged)	0.83	0.88	0.87	0.85	0.90	0.88	3.5	4.1	4.0
P26	B37	0.92	0.88	0.92	0.90	0.88	0.90	3.7	4.5	4.4
P27	B38	0.92	0.90	0.88	0.92	0.90	0.90	3.8	4.6	4.6
P28	B42	0.92	0.92	0.88	0.90	0.93	0.91	3.9	4.5	4.6

Exemplar dispositions illustrate the application of the *a priori* rules. Among verbatim retentions, K2 (originally B2) was representative, achieving a CVR of 0.92 and a CVI Total of 0.93. The revision rule was applied to K1 (originally B1), which met all numeric thresholds but was flagged by two experts for dense syntactic structure; after rewording, it was retained. The merging rule was applied to B3 and B4 (CVI Totals of 0.86 and 0.89, within the ± 0.05 threshold), which were consolidated into K3. By contrast, B11 was deemed too technically specialized for lay self-report and failed both retention thresholds (CVR = 0.42; I-CVI Total = 0.57), resulting in its removal.

The final 28-item version attained an overall CVR of 0.90 and an S-CVI/Ave of 0.91, both well above conventional thresholds. The complete audit trail for all 42 candidate items is presented in Table 1, and item-level CVR/CVI values for the retained 28 items are presented in Table 2. The full Chinese-language item-level disposition is provided in Supplementary Additional File 1.

### 3.2 Face validity

All 28 items exceeded the Item Impact Score threshold of 1.5, with scores ranging from 3.5 to 4.2 (mean = 3.86). Mean ratings for clarity and comprehensibility were 4.42 (SD = 0.31) and 4.38 (SD = 0.34), respectively. Among the items addressing culturally salient concerns in geriatric cancer pain management, item A12“I do not consider “enduring pain” to be a virtue for the elderly; expressing pain is an essential avenue for obtaining appropriate treatment” and item A13 “I agree that using opioid medications for cancer pain management, under the guidance of medical professionals, is both safe and effective, It does not lead to drug dependence or addiction” achieved Impact Scores of 3.9 and 4.0, respectively–both in the upper range of the observed distribution. These results indicate that older patients perceived items addressing pain-related stoicism and opioid phobia as understandable and personally relevant. Given this favorable evaluation, no further rewording was required for any item at this stage. Details are provided in Table 2.

### 3.3 Sociodemographic and clinical characteristics of participants

A total of 440 valid responses were obtained from 504 distributed questionnaires, yielding a valid response rate of 87.3%. The demographic, clinical, and pain-related characteristics of the participants are summarized in Table 3.

**TABLE 3:** Socio-demographic characteristics of the participants (*n* = 440).

Parameters	Categories	Total sample (*n* = 440, %)	EFA sample (*n* = 220, %)	CFA sample (*n* = 220, %)
Age	60–69	254 (57.7)	135 (61.4)	119 (54.1)
	70–79	186 (42.3)	85 (38.6)	101 (45.9)
Sex	Male	187 (42.5)	88 (40.0)	99 (45.0)
	Female	253 (57.5)	132 (60.0)	121 (55.0)
Marital status	Married	364 (82.7)	186 (84.5)	178 (80.9)
	Widowed	74 (16.8)	33 (15.0)	41 (18.6)
	Unmarried	2 (0.5)	1 (0.5)	1 (0.5)
Religious belief	Yes	42 (9.5)	26 (11.8)	16 (7.3)
	No	398 (90.5)	194 (88.2)	204 (92.7)
Residence	Rural	219 (49.8)	116 (52.7)	103 (46.8)
	City	221 (50.2)	104 (47.3)	117 (53.2)
Educational level	Illiterate	83 (18.9)	37 (16.8)	46 (20.9)
	Primary school	154 (35.0)	82 (37.3)	72 (32.7)
	Junior high school	88 (20.0)	41 (18.6)	47 (21.4)
	Senior high school	59 (13.4)	33 (15.0)	26 (11.8)
	Junior college	37 (8.4)	17 (7.7)	20 (9.1)
	Bachelor’s degree and above	19 (4.3)	10 (4.6)	9 (4.1)
Living status	Living alone	18 (4.1)	7 (3.2)	11 (5.0)
	Living with spouse and children	263 (59.8)	138 (62.7)	125 (56.8)
	Living with children	99 (22.5)	44 (20.0)	55 (25.0)
	Living with spouse	60 (13.6)	31 (14.1)	29 (13.2)
Monthly household income (RMB)	<5000	223 (50.7)	107 (48.6)	116 (52.7)
	5000–8000	130 (29.5)	71 (32.3)	59 (26.8)
	>8000	87 (19.8)	42 (19.1)	45 (20.5)
Main caregiver	Spouse	312 (70.9)	163 (74.1)	149 (67.7)
	Children	119 (27.1)	53 (24.1)	66 (30.0)
	The hired nanny	9 (2.0)	4 (1.8)	5 (2.3)
Cancer types	Digestive system tumors	216 (49.1)	103 (46.8)	113 (51.4)
	Respiratory system tumors	89 (20.2)	49 (22.3)	40 (18.2)
	Urogenital system tumors	87 (19.8)	41 (18.6)	46 (20.9)
	Hematological system tumors	48 (10.9)	27 (12.3)	21 (9.5)
Healthcare payment modalities	Urban employee basic medical insurance	191 (43.4)	89 (40.5)	102 (46.4)
	Urban and rural resident basic medical insurance	90 (20.5)	51 (23.2)	39 (17.7)
	New rural cooperative medical scheme	127 (28.8)	63 (28.6)	64 (29.1)
	Medical financial assistance for impoverished populations	32 (7.3)	17 (7.7)	15 (6.8)
Have you received any education on cancer pain management	Yes	112 (25.5)	48 (21.8)	64 (29.1)
	No	328 (74.5)	172 (78.2)	156 (70.9)
Duration of pain (months)	<6	101 (23.0)	57 (25.9)	44 (20.0)
	6∼12	141 (32.0)	64 (29.1)	77 (35.0)
	>12	198 (45.0)	99 (45.0)	99 (45.0)
The number of concurrent chronic diseases	0	48 (10.9)	20 (9.1)	28 (12.7)
	1–2	264 (60.0)	137 (62.3)	127 (57.7)
	≥3	128 (29.1)	63 (28.6)	65 (29.6)

Participants were predominantly female (57.5%), and more than half were aged 60–69 years (57.7%). Regarding cancer type, digestive system malignancies were the most frequently reported (49.1%), followed by respiratory (20.2%), urogenital (19.8%), and hematological tumors (10.9%), a distribution broadly consistent with cancer patterns observed among older adults in China. Comorbid conditions were highly prevalent: 89.1% of participants reported at least one concurrent chronic disease, and 29.1% reported three or more, underscoring the clinical complexity of this population. Pain-related characteristics indicated a substantial chronic pain burden, with 77.0% of participants reporting pain lasting at least 6 months and 45.0% reporting pain persisting for more than 12 months. These findings suggest that persistent pain was the dominant pain pattern in the sample. Notably, only 25.5% of participants had previously received education on cancer pain management, indicating limited exposure to structured pain-management information among older adults with cancer pain.

### 3.4 Construct validity

#### 3.4.1 Exploratory factor analysis (EFA)

The EFA sample (*n* = 220) met adequacy criteria (KMO = 0.94; Bartlett’s test, χ^2^ = 3688.69, *p* < 0.001). Principal component analysis with varimax rotation identified a clear three-factor structure corresponding to knowledge, attitudes, and practices. These three factors explained 61.0% of the total variance (knowledge: 37.9%, attitudes: 13.0%, practices: 10.1%). All item loadings exceeded 0.50, confirming good factor alignment without significant cross-loadings, Details are provided in Table 4.

**TABLE 4:** Factor loading matrix of KAP-Cancer Pain Geriatric.

Items	Factor loading		
	Knowledge	Attitude	Practices
1. I am familiar with the clinical features of cancer pain in older adults, including the coexistence of pain at multiple sites, atypical pain presentation, and a tendency toward chronic progression.	0.685		
2. I understand that pain is considered the “fifth vital sign” and should be treated with the same importance as other vital signs such as blood pressure and pulse, warranting regular assessment	0.732		
3. I understand that self-report and pain assessment tools specifically designed for older adults (such as numerical rating scales, facial expression scales, and similar instruments) are of significant importance in pain evaluation	0.684		
4. I understand that persistent pain can contribute to a range of problems, including physical functional decline, sleep disturbances, mood changes, and impaired social participation.	0.768		
5. I understand the critical importance of potent analgesics in geriatric cancer pain management, and their judicious application does not result in addiction	0.642		
6. I am familiar with the common side effects associated with analgesic use in the elderly, such as constipation, drowsiness, and an increased risk of falls, and I know the preventive and management strategies for these side effects	0.752		
7. I acknowledge the complementary role of various non-pharmacological pain management methods, such as heat or cold compresses and relaxation techniques, in alleviating cancer pain in the elderly	0.760		
8. I am aware of the impact of age-related physiological changes, such as diminished kidney function and reduced drug clearance, on the effectiveness and safety of pain medications	0.744		
9. I know that when pain control is inadequate, it should be reported to healthcare professionals rather than accepting pain as an inevitable consequence of aging or cancer	0.753		
10. I am knowledgeable about the relationship between analgesic dosage and factors such as age, liver and kidney function, and concurrent medications, and I understand the importance of individualized treatment plans for elderly patients	0.767		
11. I believe that effective pain management is foundational to improving the quality of life in older adults and should not be regarded as a luxury or secondary concern			0.670
12. I do not consider “enduring pain” to be a virtue for the elderly; expressing pain is an essential avenue for obtaining appropriate treatment			0.697
13. I agree that using opioid medications for cancer pain management, under the guidance of medical professionals, is both safe and effective, It does not lead to drug dependence or addiction.			0.710
14. I am willing to learn and adopt pain assessment methods that are suitable for my specific condition, to enable accurate communication of my pain experience with healthcare providers			0.753
15. I believe that pain management constitutes a fundamental patient right, and that effective pain management contributes to maintaining daily functional capacity and preserving patient autonomy and independence			0.751
16. I am open to discussing the side effects of medications with healthcare professionals, rather than silently enduring them or discontinuing the medication on my own			0.786
17. I recognize the importance of multimodal pain management (combining both pharmacological and non-pharmacological approaches) and am willing to explore integrated treatment plans			0.778
18. I understand that adjustments in pain medication dosages may be necessary, and this is a normal part of the treatment process, not an indication of drug addiction			0.728
19. I am willing to share my pain experiences with family members and accept their support and assistance in managing my pain			0.697
20. I consistently utilize standardized pain diaries or assessment scales to systematically record pain intensity, location, characteristics, and the impact of pain on my daily functional activities		0.735	
21. I adhere to a prescribed schedule for taking analgesic medications, rather than delaying administration until the pain intensifies		0.804	
22. I proactively communicate the status of my pain management to healthcare providers, including any concerns regarding insufficient efficacy and any possible adverse effects.		0.778	
23. In addition to pharmacological treatment, I employ various non-pharmacological pain management techniques (e.g., heat therapy, cold therapy, relaxation techniques) as supplementary interventions		0.719	
24. Concurrent with opioid use, I follow medical guidance in using appropriate preventive measures or medications to mitigate constipation		0.779	
25. I conduct regular assessments of my physical functional status and modify my daily activities accordingly to prevent pain exacerbation, while simultaneously avoiding excessive activity restriction		0.765	
26. I actively share my pain management plan with family members or caregivers to ensure they are well-informed and capable of providing the necessary assistance		0.801	
27. I carry emergency medication for pain at all times and strictly adhere to the prescribed dosage and administration instructions.		0.799	
28. I actively educate myself on the proper use of various drug delivery devices (e.g., transdermal patches, titration pumps) to ensure accurate medication administration and optimal pain control		0.720	
**% of explained variance**	**37.923**	**12.988**	**10.053**

Extraction method: principal component analysis. Rotation method: varimax. Kaiser-Meyer-Olkin measure of sampling adequacy: 0.943. Bartlett’s test of sphericity *p* < 0.0001 (Approx. Chi-square: 3688.689, df: 378). Bold values indicate the percentage of total variance explained by the corresponding factor.

#### 3.4.2 Confirmatory factor analysis (CFA)

**FIGURE 1:**
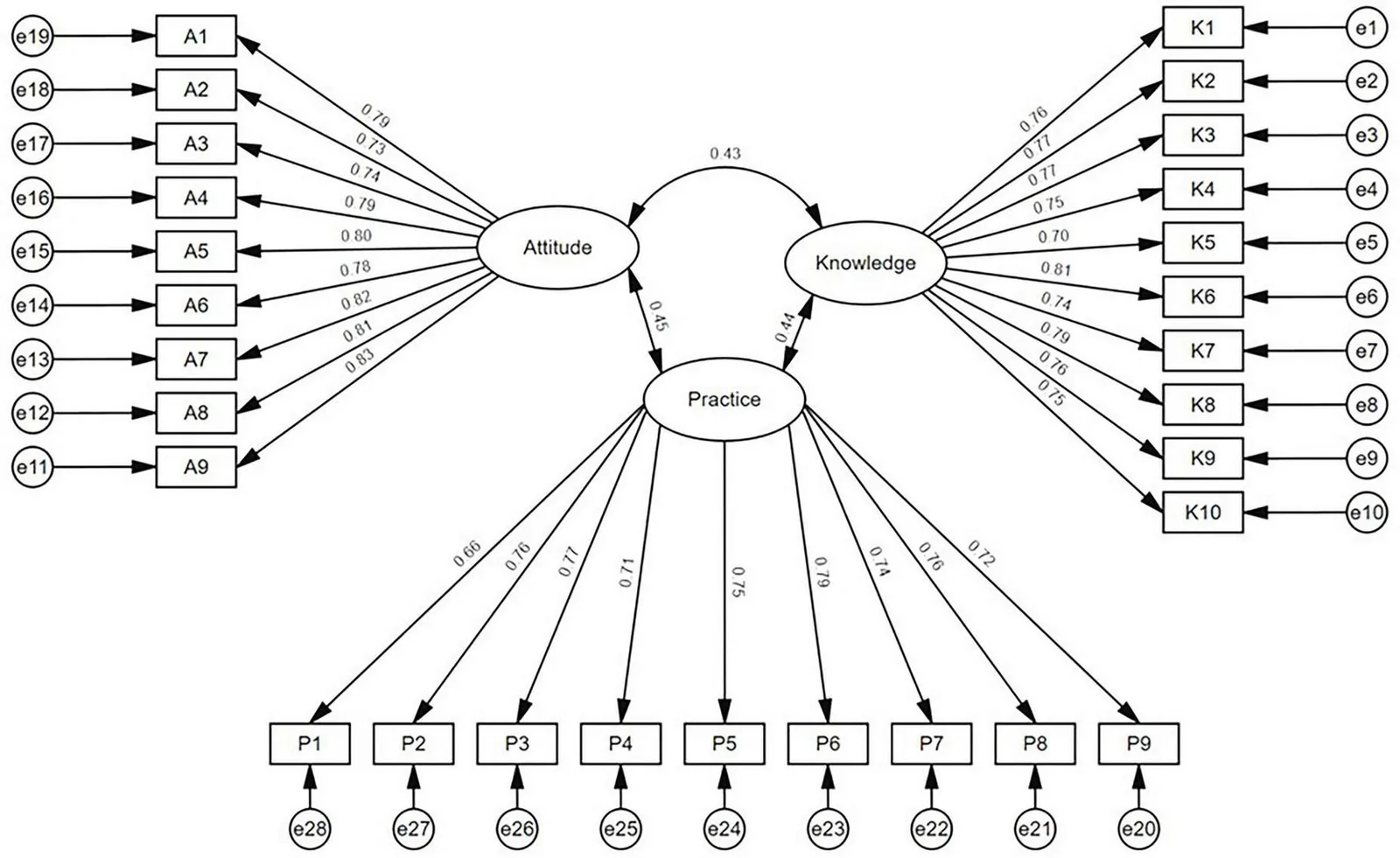
Standardized three-factor confirmatory factor analysis model of the Knowledge–Attitude–Practice Questionnaire for Geriatric Cancer Pain Management (KAP-CPGQ). For clarity, the attitude items labeled A1–A9 in the CFA model correspond to final questionnaire items A11–A19, and the practice items labeled P1–P9 correspond to final questionnaire items P20–P28; knowledge items K1–K10 retain the same numbering.

Confirmatory factor analysis conducted on the second subsample (*n* = 220) supported the three-factor model. The goodness-of-fit indices indicated excellent model fit: χ^2^/df = 1.158, AGFI = 0.869, NFI = 0.903, NNFI = 0.984, CFI = 0.986, IFI = 0.986, RMR = 0.026, and RMSEA = 0.027. Standardized factor loadings ranged from 0.66 to 0.83, demonstrating robust convergent validity. Details are provided in Figure 1.

### 3.5 Reliability analysis

The KAP-CPGQ exhibited excellent internal consistency. The overall Cronbach’s α was 0.94, while the subscales ranged from 0.92 to 0.93. Split-half reliability was 0.90 overall (subscales: 0.87–0.89). Test–retest reliability assessed after a 2-week interval (*n* = 30) ranged from 0.89 to 0.91 across the three domains, indicating good temporal stability. Details are provided in Table 5.

**TABLE 5:** Reliability analysis of the Knowledge–Attitude–Practice Questionnaire for Geriatric Cancer Pain Management.

Reliability measure	Knowledge	Attitude	Practice	Total
Cronbach’s α	0.93	0.93	0.92	0.94
Split-half reliability	0.89	0.88	0.87	0.90
Test-retest reliability	0.91	0.90	0.89	0.92

### 3.6 Final questionnaire

The finalized KAP-CPGQ consisted of 28 items across three dimensions: knowledge (10 items), attitudes (9 items), and practices (9 items). Each item was rated on a 5-point Likert scale. Higher scores indicated greater knowledge, more positive attitudes, and better pain management practices.

## 4 Discussion

This study developed and validated the Knowledge–Attitude–Practice Questionnaire for Geriatric Cancer Pain Management (KAP-CPGQ), a 28-item patient-centered instrument designed to assess cancer pain management–related knowledge, attitudes, and practices among geriatric Chinese patients with cancer. Psychometric evaluation showed that the KAP-CPGQ met accepted standards for structural validity, internal consistency, and temporal reliability for patient-reported measures (Boateng et al., 2018; Terwee et al., 2018). Beyond its psychometric performance, the instrument addresses an important gap in existing assessment tools by conceptualizing cancer pain management from the perspective of patient agency rather than provider competence. This orientation is consistent with the view that geriatric patients are not merely recipients of pain care but active participants in cancer pain control (Antunes et al., 2024; Chi et al., 2023).

### 4.1 Interpretation of psychometric findings

The exploratory factor analysis yielded a three-factor solution corresponding to the *a priori* knowledge, attitude, and practice domains. Sampling adequacy was supported by a KMO value of 0.94 and a significant Bartlett’s test, both within the ranges considered acceptable for factor extraction (Boateng et al., 2018). The three retained factors accounted for 61.0% of the total variance, with all items loading above 0.50 on their intended factors and no notable cross-loadings. These results indicate that the *a priori* domain structure was empirically supported by the data. The confirmatory factor analysis conducted on an independent split-half subsample yielded fit indices consistent with conventional benchmarks (χ^2^/df = 1.158; CFI = 0.986; NNFI = 0.984; RMSEA = 0.027; RMR = 0.026), with standardized loadings ranging from 0.66 to 0.83. Internal consistency (overall α = 0.94; subscales 0.92–0.93) is at the upper end of the acceptable range, indicating adequate item coherence without crossing the redundancy threshold of 0.95. Two-week test–retest reliability values ranging from 0.89 to 0.91 across the three subscales support temporal stability, suggesting the instrument is suitable for longitudinal monitoring. A methodological feature worth noting is the random partitioning of the 440-respondent dataset for independent EFA and CFA, which reduces the inflation that typically arises when the same sample is used for both factor extraction and confirmation (Boateng et al., 2018).

### 4.2 Cultural and patient-centered relevance

A key feature of the KAP-CPGQ is its inclusion of items specifically designed to evaluate culturally relevant factors in pain expression and analgesic use. These factors were first identified during the qualitative expert interview phase and subsequently validated and retained through the Delphi consensus process. One notable example is ren tong (“enduring pain”), a culturally endorsed valuation of pain tolerance, which can manifest among Chinese patients both as a socially normative behavior and as an internalized personal expectation (Xu et al., 2018, 2019; Chen et al., 2012). Concurrently, patients’ concerns regarding opioid use represent another critical domain, encompassing fears of medication dependence as well as anxiety about potential judgments from family members or the wider community (Xu et al., 2018, 2023). Moreover, in many Chinese families, adult children frequently participate in treatment decision-making, a role that often extends to pain disclosure and consent for analgesic administration, reflecting cultural norms of filial caregiving (Ng et al., 2024; Wu et al., 2025; Jeon and Jing, 2023).

Widely used pain assessment scales predominantly focus on specific dimensions of the pain experience. The Brief Pain Inventory evaluates pain intensity and its impact on daily functioning (Cleeland and Ryan, 1994), whereas the McGill Pain Questionnaire examines both the sensory and affective components of pain (Melzack, 1975). The Pain Attitudes Questionnaire–Revised was developed to measure pain-related attitudes; however, its development and validation were confined to North American populations with advanced cancer (Mah et al., 2017), and its applicability to Chinese patients has not been established. Research involving patients in mainland China and overseas Chinese communities has identified several culturally influenced barriers to adequate analgesic use, including fatalistic beliefs, fear of opioid dependence, and reluctance to report pain (Xu et al., 2018; Zeng et al., 2020; Edrington et al., 2009). Supporting this, the multinational ACHEON survey reported that 53% of physicians in China identified patients’ fear of addiction as a major impediment to effective pain management, while 43% cited patients’ unwillingness to report pain as a primary barrier (Kim et al., 2015; Xia, 2017). In this context, the KAP-CPGQ provides a culturally sensitive tool for assessing these patient-level factors, addressing a gap not captured by existing instruments.

### 4.3 Clinical implications

In clinical practice, the KAP-CPGQ has several potential applications. It may be used to identify geriatric patients with limited pain-related knowledge, attitudinal barriers, or inadequate self-management behaviors that could compromise analgesic effectiveness. These barriers may include an excessive emphasis on enduring pain, concerns about opioid use, and irregular medication-taking practices. Beyond screening, the scale can also inform individualized health education and clinical communication. For instance, a patient with a low attitude-subscale score driven by opioid-related concerns would require a different counseling approach from a patient with a low practice-subscale score resulting from inconsistent scheduled dosing. In addition, because patient-based educational interventions for cancer pain have been shown to produce modest but statistically significant reductions in pain intensity and improvements in patients’ knowledge and attitudes (Bennett et al., 2009), the KAP-CPGQ may provide a practical outcome measure for evaluating patient-education programmes in routine care settings.

From a service-organization perspective, a brief and reliable patient-level KAP measure for cancer pain management in geriatric patients could be readily incorporated into team-based supportive-care models. These models commonly bring together oncology, geriatric medicine, palliative care, and nursing to address the multidimensional needs of geriatric patients with cancer. For geriatric Chinese patients with cancer, particularly those with multimorbidity and frailty, such integrated care is especially relevant because pain management often requires coordinated assessment, communication, medication monitoring, and ongoing patient and family education.

### 4.4 Strengths and limitations

A major strength of this study is its rigorous multi-phase design, incorporating qualitative item generation, expert Delphi consultation, and large-sample psychometric testing. The integration of exploratory and confirmatory factor analyses enhances construct validity and ensures the instrument’s stability.

However, the study has limitations. Participants were recruited from tertiary hospitals within a single province, which may limit generalizability. Broader multi-center validation is warranted to confirm applicability across diverse regions and healthcare settings. Additionally, self-report data may be influenced by social desirability bias. Future studies could complement quantitative assessment with qualitative approaches to explore deeper psychosocial dimensions of pain management among older adults.

### 4.5 Future directions

Future research should focus on longitudinal validation and intervention applications of the KAP-CPGQ. The tool could be integrated into patient education programs or supportive care pathways to evaluate changes in knowledge, attitudes, and self-management behaviors over time. Cross-cultural validation among other East Asian populations may also strengthen its international utility. Ultimately, applying this tool in clinical settings can help bridge the gap between patient beliefs and effective pain control, supporting more culturally sensitive and patient-driven care strategies.

## 5 Conclusion

The Knowledge–Attitude–Practice Questionnaire for Geriatric Cancer Pain Management (KAP-CPGQ) is a reliable, valid, and culturally adapted instrument that captures the multidimensional aspects of pain management among older adults with cancer. By centering on patients’ perspectives and incorporating culturally relevant psychosocial factors, it fills a critical gap in existing assessment tools. The KAP-CPGQ offers clinicians and researchers a valuable resource for evaluating pain-related beliefs and behaviors, guiding targeted interventions, and ultimately improving the quality of life of geriatric cancer patients through enhanced supportive care.

## Data Availability

The raw data supporting the conclusions of this article will be made available by the authors, without undue reservation.
