# Supplementary material for: Development and psychometric validation of a patient-centered Knowledge, Attitude, and Practice (KAP) Questionnaire for Geriatric Cancer Pain Management
Source: Front Psychol. 2026 Sep 9;17:1808383. doi: 10.3389/fpsyg.2026.1808383 (PMC13559704; doi:10.3389/fpsyg.2026.1808383)
Supplement: Supplementary file 1 [file Supplementary_file_1.docx]

# Supplementary Additional File 1. Full 42-Item Disposition Tracking Table

| Original ID | Domain | Original Wording  (Chinese) | English Translation | CVR | CVI  (Relevance) | CVI  (Clarity) | CVI  (Simplicity) | CVI  (Ambiguity) | CVI  (Total) | Disposition | Reason | Final Wording after Revision / Merging |
| --- | --- | --- | --- | --- | --- | --- | --- | --- | --- | --- | --- | --- |
|  |  |  |  |  |  |  |  |  |  |  |  |  |
| B1 | K | 我了解老年人癌性疼痛的临床特征，主要表现为多部位疼痛并存、疼痛症状表达不典型以及疼痛慢性化风险增加等。 | I am familiar with the clinical characteristics of cancer pain in the elderly, which primarily include the coexistence of pain in multiple sites, atypical expression of pain symptoms, and an increased risk of chronic pain. | 0.83 | 0.92 | 0.90 | 0.88 | 0.93 | 0.91 | Revised → K1 | Wording simplified for elderly readers | 我了解老年人癌性疼痛的特点，如存在多个部位疼痛或疼痛不典型，逐渐表现为慢性疼痛的特点等。  I am familiar with the clinical features of cancer pain in older adults, including the coexistence of pain at multiple sites, atypical pain presentation, and a tendency toward chronic progression. |
| B2 | K | 我知道疼痛是“第五项生命体征”，应当与血压、脉搏等指标一样被重视和评估。 | I understand that pain is considered the "fifth vital sign" and should be treated with the same importance as other vital signs such as blood pressure and pulse, warranting regular assessment. | 0.92 | 0.95 | 0.92 | 0.90 | 0.95 | 0.93 | Retained → K2 | Met all thresholds |  |
| B3 | K | 我了解自我报告是评估疼痛的金标准，即使我有认知障碍，我的疼痛表达也应被医务人员重视。 | I recognize that self-reporting is the gold standard for pain assessment, and even in cases of cognitive impairment, the expression of pain should be taken seriously by healthcare professionals. | 0.83 | 0.85 | 0.88 | 0.82 | 0.90 | 0.86 | Merged with B4 → K3 | Conceptual overlap on self-report assessment | 【合并B4后】我了解自我报告以及专为老年人设计的疼痛评估工具（如数字评定量表、面部表情量表等）在疼痛评估中具有重要意义。  [Merged B4] I understand that self-report and pain assessment tools specifically designed for older adults (such as numerical rating scales, facial expression scales, and similar instruments) are of significant importance in pain evaluation. |
| B4 | K | 我掌握适合老年人使用的疼痛评估工具（如数字评定量表、面部表情量表等）及其适用场景。 | I am knowledgeable about pain assessment tools appropriate for the elderly (such as the numeric rating scale , Faces Pain Scale, etc.), as well as the contexts in which these tools are most applicable. | 0.83 | 0.90 | 0.88 | 0.85 | 0.92 | 0.89 | Merged with B3 → K3 | Conceptual overlap on self-report assessment | 【合并 B3后】我了解自我报告以及专为老年人设计的疼痛评估工具（如数字评定量表、面部表情量表等）在疼痛评估中具有重要意义。  [Merged B3] I understand that self-report and pain assessment tools specifically designed for older adults (such as numerical rating scales, facial expression scales, and similar instruments) are of significant importance in pain evaluation. |
| B5 | K | 我知道长期未控制的疼痛会导致身体功能下降、抑郁、睡眠障碍和社会隔离等老年特有并发症。 | I know that poorly controlled pain over time can lead to age-specific complications such as physical decline, depression, sleep disturbances, and social isolation. | 0.83 | 0.90 | 0.88 | 0.85 | 0.90 | 0.88 | Revised → K4 | Disambiguation of geriatric-specific complications | 我知道长期疼痛会导致身体功能下降、睡眠障碍、情绪变化、不能参与社会活动等问题。  I understand that persistent pain can contribute to a range of problems, including physical functional decline, sleep disturbances, mood changes, and impaired social participation. |
| B6 | K | 我了解阿片类药物在老年癌痛管理中的重要性及其与疾病进展无关的科学事实。 | I understand the importance of opioid medications in cancer pain management for the elderly and the scientific fact that their use is not associated with disease progression. | 0.83 | 0.90 | 0.85 | 0.88 | 0.92 | 0.89 | Merged with B7 → K5 | Conceptual overlap on opioid safety | 【合并B7后】我了解强效的止痛药在老年癌痛管理中的重要性，合理使用不会导致成瘾  [Merged B7] I understand the critical importance of potent analgesics in geriatric cancer pain management, and their judicious application does not result in addiction |
| B7 | K | 我知道在科学监督下合理使用阿片类药物不会导致成瘾，风险远低于疼痛未控带来的危害。 | I know that when used appropriately under medical supervision, opioid medications do not lead to addiction, and the risks are far lower than the harm caused by uncontrolled pain. | 0.83 | 0.92 | 0.88 | 0.90 | 0.93 | 0.91 | Merged with B6 → K5 | Conceptual overlap on opioid safety | 【合并B6后】我了解强效的止痛药在老年癌痛管理中的重要性，合理使用不会导致成瘾  [Merged B6] I understand the critical importance of potent analgesics in geriatric cancer pain management, and their judicious application does not result in addiction |
| B8 | K | 我知晓老年人镇痛药物治疗中常见副作用（便秘、嗜睡、跌倒风险等）及其预防和处理方法。 | I am familiar with the common side effects associated with analgesic use in the elderly, such as constipation, drowsiness, and an increased risk of falls, and I know the preventive and management strategies for these side effects. | 0.92 | 0.92 | 0.95 | 0.90 | 0.93 | 0.93 | Retained → K6 | Met all thresholds |  |
| B9 | K | 我理解多种非药物镇痛方法（如热敷、冷敷、放松训练等）对管理老年癌痛的辅助作用。 | I acknowledge the complementary role of various non-pharmacological pain management methods, such as heat or cold compresses and relaxation techniques, in alleviating cancer pain in the elderly. | 0.92 | 0.93 | 0.90 | 0.92 | 0.95 | 0.93 | Retained → K7 | Met all thresholds |  |
| B10 | K | 我了解老年期特有的生理变化（如肾功能下降、药物清除率降低）对疼痛药物使用的影响。 | I am aware of the impact of age-related physiological changes, such as diminished kidney function and reduced drug clearance, on the effectiveness and safety of pain medications. | 0.92 | 0.90 | 0.88 | 0.85 | 0.92 | 0.89 | Retained → K8 | Met all thresholds |  |
| B11 | K | 我熟悉癌性爆发痛的临床表现特征及其处理原则，包括速效阿片类药物的合理使用时机与给药方法。 | I am familiar with the clinical characteristics and management principles of cancer breakthrough pain, including the appropriate use and administration timing of fast-acting opioids. | 0.42 | 0.55 | 0.62 | 0.50 | 0.62 | 0.57 | Deleted | Excessive technical complexity unsuitable for lay self-report |  |
| B12 | K | 我了解老年人多重慢性病共存时各类镇痛药物潜在的相互作用和注意事项。 | I understand the potential drug interactions and precautions to consider when elderly patients are being treated with analgesics, particularly in the presence of multiple chronic conditions. | 0.50 | 0.65 | 0.70 | 0.68 | 0.75 | 0.69 | Deleted | Conceptual redundancy with K6 |  |
| B13 | K | 我知晓疼痛控制不理想时应该向医护人员报告，而非认为疼痛是老年人或癌症不可避免的结局。 | I know that when pain control is inadequate, it should be reported to healthcare professionals rather than accepting pain as an inevitable consequence of aging or cancer. | 0.92 | 0.95 | 0.92 | 0.88 | 0.93 | 0.92 | Retained → K9 | Met all thresholds |  |
| B14 | K | 我了解镇痛药物剂量与年龄、肝肾功能、合并用药等因素的关系，以及为何老年人需要个体化给药方案。 | I am knowledgeable about the relationship between analgesic dosage and factors such as age, liver and kidney function, and concurrent medications, and I understand the importance of individualized treatment plans for elderly patients. | 0.92 | 0.88 | 0.92 | 0.90 | 0.95 | 0.91 | Retained → K10 | Met all thresholds |  |
| B15 | A | 我认为良好的疼痛控制是提高老年人生活质量的基础，不应视为奢侈或次要需求。 | I believe that effective pain management is foundational to improving the quality of life in older adults and should not be regarded as a luxury or secondary concern. | 0.92 | 0.95 | 0.90 | 0.92 | 0.88 | 0.91 | Retained → A11 | Met all thresholds |  |
| B16 | A | 我不认为“忍痛”是老年人应有的美德，疼痛表达是获得适当治疗的必要途径。 | I do not consider "enduring pain" to be a virtue for the elderly; expressing pain is an essential avenue for obtaining appropriate treatment. | 0.92 | 0.90 | 0.93 | 0.88 | 0.92 | 0.91 | Retained → A12 | Met all thresholds |  |
| B17 | A | 我认同在规范医疗指导下使用阿片类药物治疗癌痛是安全有效的，既不会导致药物依赖或成瘾问题。 | I agree that using opioid medications for cancer pain management, under the guidance of medical professionals, is both safe and effective. It does not lead to drug dependence or addiction. | 0.92 | 0.92 | 0.88 | 0.90 | 0.95 | 0.91 | Retained → A13 | Met all thresholds |  |
| B18 | A | 我愿意学习并掌握适合自身情况的疼痛评估方法，以便与医护人员准确沟通我的疼痛体验。 | I am willing to learn and adopt pain assessment methods that are suitable for my specific condition, to enable accurate communication of my pain experience with healthcare providers. | 0.92 | 0.88 | 0.90 | 0.92 | 0.90 | 0.90 | Retained → A14 | Met all thresholds |  |
| B19 | A | 我认为疼痛管理是我作为患者的优先权利，不应因年龄大而降低治疗期望。 | I believe that pain management is a fundamental right for patients, and treatment expectations should not be diminished based on age. | 0.83 | 0.88 | 0.85 | 0.85 | 0.85 | 0.86 | Merged with B20 → A15 | Conceptual overlap on patient-rights orientation | 【合并B20后】我认为疼痛管理是患者的权利，正确管理疼痛助于维持日常功能和独立自主能力。  [Merged B20] I believe that pain management constitutes a fundamental patient right, and that effective pain management contributes to maintaining daily functional capacity and preserving patient autonomy and independence |
| B20 | A | 我认为，正确管理疼痛不仅不会增加依赖性，反而有助于维持我的日常功能和独立自主能力。 | I believe that actively managing pain helps maintain my daily functioning and independence, rather than increasing my reliance on others. | 0.83 | 0.90 | 0.85 | 0.88 | 0.87 | 0.88 | Merged with B19 → A15 | Conceptual overlap on patient-rights orientation | 【合并B19后】我认为疼痛管理是患者的权利，正确管理疼痛助于维持日常功能和独立自主能力。  [Merged B19] I believe that pain management constitutes a fundamental patient right, and that effective pain management contributes to maintaining daily functional capacity and preserving patient autonomy and independence |
| B21 | A | 我愿意就药物副作用与医务人员坦诚沟通，而非默默忍受或自行停药。 | I am open to discussing the side effects of medications with healthcare professionals, rather than silently enduring them or discontinuing the medication on my own. | 0.92 | 0.90 | 0.92 | 0.88 | 0.93 | 0.91 | Retained → A16 | Met all thresholds |  |
| B22 | A | 我认同多模式疼痛管理（药物结合非药物方法）的重要性，愿意尝试综合治疗方案。 | I recognize the importance of multimodal pain management (combining both pharmacological and non-pharmacological approaches) and am willing to explore integrated treatment plans. | 0.92 | 0.93 | 0.88 | 0.90 | 0.92 | 0.91 | Retained → A17 | Met all thresholds |  |
| B23 | A | 我不认为疼痛加剧一定意味着癌症进展，它可能与多种因素有关，需专业评估。 | I do not believe that an increase in pain necessarily indicates cancer progression; pain exacerbation may result from various factors and requires professional evaluation. | 0.50 | 0.62 | 0.70 | 0.68 | 0.72 | 0.68 | Deleted | Limited cultural applicability; outside KAP framework |  |
| B24 | A | 我理解癌痛药物治疗中可能需要剂量调整，这属于正常治疗过程而非药物成瘾的表现。 | I understand that adjustments in pain medication dosages may be necessary, and this is a normal part of the treatment process, not an indication of drug addiction. | 0.92 | 0.92 | 0.90 | 0.88 | 0.95 | 0.91 | Retained → A18 | Met all thresholds |  |
| B25 | A | 我愿意与家庭成员分享我的疼痛体验，接受他们在疼痛管理中的支持和协助。 | I am willing to share my pain experiences with family members and accept their support and assistance in managing my pain. | 0.92 | 0.88 | 0.92 | 0.90 | 0.88 | 0.90 | Retained → A19 | Met all thresholds |  |
| B26 | A | 我认为即使在癌症晚期，维持良好的疼痛控制也是必要的生活质量保障。 | I believe that maintaining effective pain control is crucial to ensuring quality of life, even in the advanced stages of cancer. | 0.42 | 0.58 | 0.65 | 0.62 | 0.68 | 0.63 | Deleted | Conceptual redundancy with A11 |  |
| B27 | A | 我相信跨专业团队（医生、护士、药师、康复师等）合作能提供更全面的疼痛管理。 | I trust that a multidisciplinary approach involving doctors, nurses, pharmacists, and rehabilitation therapists can provide a more comprehensive and effective pain management strategy. | 0.50 | 0.62 | 0.68 | 0.65 | 0.70 | 0.66 | Deleted | Insufficient specificity to pain self-management |  |
| B28 | A | 我认同定期随访和持续评估对优化老年癌痛管理方案的重要性。 | I acknowledge that regular follow-ups and ongoing assessments are critical for optimizing cancer pain management in the elderly. | 0.50 | 0.65 | 0.70 | 0.62 | 0.72 | 0.67 | Deleted | Conceptual redundancy with B30 |  |
| B29 | A | 我不将服用镇痛药视为软弱或无法承受的表现，而是科学管理健康的积极行为。 | I do not perceive the use of analgesic medications as a sign of weakness or an inability to cope, but rather as a responsible and proactive approach to managing my health. | 0.42 | 0.55 | 0.60 | 0.58 | 0.65 | 0.60 | Deleted | Limited cultural applicability |  |
| B30 | P | 我会定期使用规范的疼痛日记或评估量表，系统记录自己的疼痛强度、疼痛部位、疼痛性质以及疼痛对患者日常生活活动的影响程度。 | I consistently utilize standardized pain diaries or assessment scales to systematically record pain intensity, location, characteristics, and the impact of pain on my daily functional activities. | 0.92 | 0.92 | 0.90 | 0.88 | 0.93 | 0.91 | Retained → P20 | Met all thresholds |  |
| B31 | P | 我按照医嘱定时服用镇痛药物，而非等到疼痛加重才服用。 | I adhere to a prescribed schedule for taking analgesic medications, rather than delaying administration until the pain intensifies | 0.92 | 0.90 | 0.92 | 0.88 | 0.90 | 0.90 | Retained → P21 | Met all thresholds |  |
| B32 | P | 我主动向医护人员报告疼痛控制情况，包括疗效不佳和可能的副作用。 | I proactively communicate the status of my pain management to healthcare providers, including any concerns regarding insufficient efficacy and any possible adverse effects. | 0.92 | 0.88 | 0.90 | 0.92 | 0.88 | 0.90 | Retained → P22 | Met all thresholds |  |
| B33 | P | 我使用多种非药物疼痛管理方法（如热敷、冷敷、放松技术等）辅助药物治疗。 | In addition to pharmacological treatment, I employ various non-pharmacological pain management techniques (e.g., heat therapy, cold therapy, relaxation techniques) as supplementary interventions. | 0.92 | 0.93 | 0.88 | 0.90 | 0.92 | 0.91 | Retained → P23 | Met all thresholds |  |
| B34 | P | 我服用阿片类药物的同时，按医嘱同步使用预防便秘的药物或措施。 | Concurrent with opioid use, I follow medical guidance in using appropriate preventive measures or medications to mitigate constipation. | 0.92 | 0.90 | 0.88 | 0.92 | 0.90 | 0.90 | Retained → P24 | Met all thresholds |  |
| B35 | P | 我根据自身情况调整日常活动，避免加重疼痛但也不过度限制活动。 | I adjust my daily activities based on my current condition, ensuring a balance between avoiding pain exacerbation and preventing unnecessary restrictions on my activities. | 0.83 | 0.88 | 0.85 | 0.85 | 0.88 | 0.87 | Merged with B36 → P25 | Conceptual overlap on activity self-regulation | 【合并B36后】我定期评估自己的身体功能状态，根据自身情况调整日常活动，避免加重疼痛，但也不过度限制活动。  [Merged B36] I conduct regular assessments of my physical functional status and modify my daily activities accordingly to prevent pain exacerbation, while simultaneously avoiding excessive activity restriction |
| B36 | P | 我定期评估自己的身体功能状态，观察疼痛对日常生活能力的影响。 | I regularly evaluate my functional status and monitor the effects of pain on my ability to perform daily tasks. | 0.83 | 0.88 | 0.87 | 0.85 | 0.90 | 0.88 | Merged with B35 → P25 | Conceptual overlap on activity self-regulation | 【合并B35后】我定期评估自己的身体功能状态，根据自身情况调整日常活动，避免加重疼痛，但也不过度限制活动。  [Merged B35] I conduct regular assessments of my physical functional status and modify my daily activities accordingly to prevent pain exacerbation, while simultaneously avoiding excessive activity restriction |
| B37 | P | 我与家人或照顾者分享我的疼痛管理计划，确保他们了解如何协助我。 | I actively share my pain management plan with family members or caregivers to ensure they are well-informed and capable of providing the necessary assistance. | 0.92 | 0.88 | 0.92 | 0.90 | 0.88 | 0.90 | Retained → P26 | Met all thresholds |  |
| B38 | P | 我会随身携带用于缓解疼痛的应急药物，并严格按照医嘱的剂量和方法正确使用。 | I carry emergency medication for pain at all times and strictly adhere to the prescribed dosage and administration instructions. | 0.92 | 0.90 | 0.88 | 0.92 | 0.90 | 0.90 | Retained → P27 | Met all thresholds |  |
| B39 | P | 我在就诊前准备疼痛相关信息（如疼痛日记），以便与医生进行有效沟通。 | Prior to medical appointments, I prepare relevant pain-related information (such as pain diaries) to facilitate effective communication with my physician. | 0.50 | 0.65 | 0.72 | 0.68 | 0.74 | 0.70 | Deleted | Conceptual redundancy with P26 |  |
| B40 | P | 我采取措施预防或管理镇痛药物可能的副作用（如补充水分、增加适当运动等）。 | I implement strategies to prevent or manage potential side effects of analgesic medications, such as maintaining adequate hydration and engaging in appropriate physical activity. | 0.42 | 0.55 | 0.62 | 0.60 | 0.68 | 0.61 | Deleted | Insufficient specificity |  |
| B41 | P | 我根据疼痛强度使用不同的应对策略，合理分配精力完成必要的日常活动。 | I employ a range of coping strategies based on pain intensity, efficiently allocating energy to complete essential daily activities. | 0.50 | 0.62 | 0.70 | 0.65 | 0.72 | 0.67 | Deleted | Limited cultural applicability |  |
| B42 | P | 我主动学习并正确使用各类给药装置（如贴剂、滴定泵等），确保用药准确。 | I actively educate myself on the proper use of various drug delivery devices (e.g., transdermal patches, titration pumps) to ensure accurate medication administration and optimal pain control. | 0.92 | 0.92 | 0.88 | 0.90 | 0.93 | 0.91 | Retained → P28 | Met all thresholds |  |
